# Optimal selective floor cleaning using deep learning algorithms and reconfigurable robot hTetro

**DOI:** 10.1038/s41598-022-19249-7

**Published:** 2022-09-24

**Authors:** Balakrishnan Ramalingam, Anh Vu Le, Zhiping Lin, Zhenyu Weng, Rajesh Elara Mohan, Sathian Pookkuttath

**Affiliations:** 1 ROAR Lab, Engineering Product Development, Singapore University of Technology and Design, Singapore, 487372 Singapore; 2 School of Electrical and Electronics Engineering, Nanyang Technological University, Singapore, 639798 Singapore

**Keywords:** Engineering, Electrical and electronic engineering

## Abstract

Floor cleaning robots are widely used in public places like food courts, hospitals, and malls to perform frequent cleaning tasks. However, frequent cleaning tasks adversely impact the robot’s performance and utilize more cleaning accessories (such as brush, scrubber, and mopping pad). This work proposes a novel selective area cleaning/spot cleaning framework for indoor floor cleaning robots using RGB-D vision sensor-based Closed Circuit Television (CCTV) network, deep learning algorithms, and an optimal complete waypoints path planning method. In this scheme, the robot will clean only dirty areas instead of the whole region. The selective area cleaning/spot cleaning region is identified based on the combination of two strategies: tracing the human traffic patterns and detecting stains and trash on the floor. Here, a deep Simple Online and Real-time Tracking (SORT) human tracking algorithm was used to trace the high human traffic region and Single Shot Detector (SSD) MobileNet object detection framework for detecting the dirty region. Further, optimal shortest waypoint coverage path planning using evolutionary-based optimization was incorporated to traverse the robot efficiently to the designated selective area cleaning/spot cleaning regions. The experimental results show that the SSD MobileNet algorithm scored 90% accuracy for stain and trash detection on the floor. Further, compared to conventional methods, the evolutionary-based optimization path planning scheme reduces 15% percent of navigation time and 10% percent of energy consumption.

## Introduction

Generally, human traffic is heavy on commercial premises like shopping malls, food courts, community clubs, and hospitals. Therefore, the chances of accumulating trash, stains (footprint stains and other forms of stains), and debris are quite high, raising safety and hygiene concerns. Hence, frequent cleaning is essential in commercial premises in pandemic situations like COVID-19. Due to long working hours, low wages, and unwillingness to work as a cleaner, workforce shortage has been a constant problem for cleaning and maintenance tasks in commercial premises^1,2^.

In recent years, mobile cleaning robots have been widely used to clean and maintain commercial premises and help overcome the workforce shortage. It performs the cleaning task in public premises using two cleaning modes: auto cleaning/scheduled cleaning mode and selective area cleaning/spot cleaning mode^2–5^. Here, the auto cleaning/scheduled cleaning mode cleans the given workspace autonomously once a day or at a scheduled time interval using a pre-build floor map. In selective area cleaning/spot cleaning mode, the robot will clean only dirty areas instead of cleaning the whole region. In contrast with auto cleaning/scheduled cleaning mode, the selective area cleaning/spot cleaning mode could save energy by avoiding non-dirty areas, reducing the cleaning time, and enhancing the lifetime of the robot^6^. However, the selective area cleaning/spot cleaning mode hasn’t been designed with autonomous operation, where the robot requires human assistance to perform the selective area cleaning/spot cleaning. Moreover, human-assisted selective area cleaning or spot cleaning is labor-intensive and time-consuming for larger workspaces. The cleaning supervisor needs to continuously monitor or inspect the environment to enable selective area cleaning/spot cleaning mode (according to human traffic in the environment or trash and stains detection).

Computer vision-based autonomous selective area cleaning/spot cleaning is a viable solution for overcoming the shortcomings mentioned above. Recently, Machine Learning (ML) and Deep Learning (DL) based computer vision algorithms are widely used for mobile cleaning robots for stain and trash detection and path planning tasks^7–9^. However, selective area cleaning/spot cleaning using onboard robot vision systems and generating the optimal path generation has a lot of practical challenges. Searching and finding the dirty region itself increases the energy consumption and processing time by scanning the entire area of operation. Further, monitoring human traffic from a robot perspective and computing the selective area cleaning/spot cleaning region took more computation time and power.

Hence, there is a need for a novel and scalable approach to overcome the challenges mentioned above in the selective area cleaning/spot cleaning method. Therefore, this article proposes a novel selective area cleaning/spot cleaning framework for indoor floor cleaning robots using RGB-D vision sensor based Closed Circuit Television (CCTV) network, deep learning algorithm, and optimal complete waypoints path planning algorithm. Furthermore, the main contributions of this paper are divided threefold:

- Design and development of selective area cleaning/spot cleaning using deep learning-based computer vision algorithm and RGB-D vision sensor network. The deep learning algorithms find the selective area cleaning/spot cleaning region based on detecting stains and trash on the floor and tracing the human traffic patterns (common space: hall, entrance, and hallway/entryway).
- Compute the 3D location of stain and trash in world coordinates to estimate the complete waypoint coverage path planning by evolutionary methods to drive the cleaning robot to a selective area cleaning/spot cleaning.
- Validate the efficiency of selective area cleaning/spot cleaning framework in the real workspace with in-house developed reconfigurable floor cleaning robot hTetro.

The manuscript is organized as follows—section “Related work” elaborates the related works, and the proposed system is presented in section “Overview of the proposed system”. Finally, section “Experimental results” provides the experimental results, followed by the conclusion and remarks of this work.

## Related work

This section presents the various dirt, stain, and trash detection schemes reported in the literature with detail summery (Table 1) and the optimal path planning methods used for cleaning robots. In^10^ Andersen et al. proposed a floor cleaning robot with a cleanness sensor using a computer vision algorithm and LED illumination. The sensor identifies dirty areas by analyzing surface images and producing pixel-by-pixel cleanness maps, applying a multivariable statistical approach. Borman et al.^11,12^ proposed a spectral residual filtering-based method for dirt detection. The dust threshold for various floor patterns is fixed based on maximal and minimal filter response. However, the dirt detection ratio is varied upon surface types. Mud and dirt on the floor are identified and classified for cleaning robots using Maximally Stable Extremal Regions (MSER) and Minimal Canny edge detection algorithms^13^. Lee and Banerjee^14^ suggested a solution for the overall cleaning schedule, which also generates an effective path for each cleaning cycle. Simulation-based Optimisation (SO) technique is applied for floor cleaning to avoid redundant paths and maintain tolerable dirt particle levels. In^6^ vision-based dirt detection was proposed for floor cleaning robot hTetro. Here, the authors use a periodic pattern detection filter to identify the dirt region on the floor and a tilling algorithm for generating the cleaning pattern. Grunauer et al.^15^ proposed an algorithm to detect dust spots based on Gaussian Mixture Model (GMM) applied to RGB images. This supports distinguishing the dust spots from the floor surface. Martinez et al.^16^ proposed a vision-based dust detection technique for the planar surface. The median filter and the thresholding technique were applied to distinguish the dust and the floor surface. Further, RANSAC algorithm was adopted to detect plane surfaces. However, as mentioned above, the scheme has significant practical limitations; for instance, the heavy detection of dirt depends on the floor background and cannot distinguish the dirt classes like trash or stain^11–13^.

Deep Learning (DL) based object detection is an emerging technique. Nowadays, it has been widely utilized in the autonomous robot vision system, abandoned object detection in surveillance applications^8,9^, human and vehicle tracking applications^17,18^, to detect stains and trash in cleaning robots, classify garbage in waste management. In^19^ author evaluate the efficiency of various deep learning-based object detection framework for marine trash detection. The authors report that the Single Shot Detector (SSD) architecture has high detection accuracy than the Yolo v2 and Tiny Yolo object detection models. GoogLeNet-Overfeat object detection model was trained for outdoor trash detection proposed by Rad et al.^20^. Here, the detection framework was trained with 18000 trash images containing waste papers, metal/plastic cans, and leaves. The trained model obtained a maximum of 77.3 % precision for detecting trash on the street. Ramalingam et al.^7^ proposed a debris detection and classification framework for floor cleaning robot application. The authors^7^ train the SSD MobileNet object detection algorithm with debris image dataset to detect and classify the trash and stains on the floor. Zhihong et al.,^21^ proposed an automatic trash sorting system using Region Proposal Network (RPN) and VGG-16 deep learning model. The authors report that the trained model had 3% miss detection and 9% false detection. Gaurav et al.^21^, proposed a smartphone app-based outdoor garbage localization technique where AlexNet CNN was trained to detect the outdoor garbage and detect the outdoor trash with $87\%$ detection accuracy. Yin et al.^2^ proposed Human Support Robot (HSR) based on deep learning for cleaning the tables. A darknet framework-based 16-layer CNN framework was developed. The model achieved 95% accuracy in detecting food strains and trash on the surface of the table. Further, a similar autonomous robot was developed using deep learning architecture, which has the capability to produce two-dimension trajectories from human kinesthetic instances^22,23^.

Apart from dirt, stain, and trash detection, path planning is also a key function for floor cleaning robots to achieve efficient cleaning. In the literature, path planning for cleaning robots was widely studied. Generally, spiral and zigzag patterns are widely used to generate the path for cleaning robots, aiming to maximize the area’s coverage. However, these patterns have significant limitations in covering the constrained areas, such as narrow space, and are only suitable for fixed morphology platforms^7^. Further, computing the optimal shortest path is another key challenge for the selective area cleaning/spot cleaning task. Reconfigurable morphology robots can cover the maximum area and arbitrary workspace compared with a fixed morphology robot and are more suitable for selective/ spot-cleaning tasks. Self-reconfigurable robots have attracted significant attention, and the development of autonomy systems mostly focused on autonomous motion control and mechanism design. There are only a few works in path planning for self-reconfigurable robots, e.g., the work in^24^ proposes a complete coverage path planning model trained using deep reinforcement learning (RL) for the tetromino based reconfigurable robot platform to simultaneously generate the optimal set of shapes for any pretrained. Research on path planning so far focuses on fixed-morphology robots and considered robots as a single point. But, representative path planning algorithms can be broadly classified as classical graph search algorithms (Dijkstra, A*, D*, and D* Lite)^25–28^, bio-inspired search (genetic algorithm, ant colony optimization, and particle swarm optimization)^29–35^, geometric algorithms (probabilistic road map, visibility graph, rapid-exploring random tree, cell decomposition, Voronoi graph)^36–41^. However, selective area cleaning/spot cleaning path planning for re-configurable floor cleaning robots is a new approach. It has not been explored yet. This research describes the optimal shortest waypoint coverage path planning method using evolutionary-based optimization technique for re-configurable floor cleaning robot hTetro to execute the selective area cleaning/spot cleaning task.

**Table 1:** Summary of related work.

Author	Algorithm	Application
Bormann et al.^11,12^	Spectral residual filter	Dirt and Mud detection on floor
Andersen et al.^10^	multivariable statistical approach	Dirt detection
Milinda et al.^13^	Spectral residual filter + Maximally Stable Extremal Regions	Dirt and mud detection
Martinez et al.^16^	median filter and the thresholding technique	Dust detection
Bala et al.^6^	periodic pattern detection filter	Dirt region mapping
Zhihong et al.,^21^	(RPN) and VGG-16	Trash sorting
Rad et al.^20^	OverFeat GoogleNet	Trash detection
Gaurav et al.^21^	AlexNet	Trash detection
Ramalingam et al.^7^	SSD MobileNet	Debris detection and classification
Yin et al.^2^	16 layer CNN	Food strains and trash detection
Chen et al.^21^	Fast RCNN	Trash sorting
Proposed system	Deep SORT and SSD MobileNet	Selective area cleaning/spot cleaning

## Overview of the proposed system

**Figure 1:**
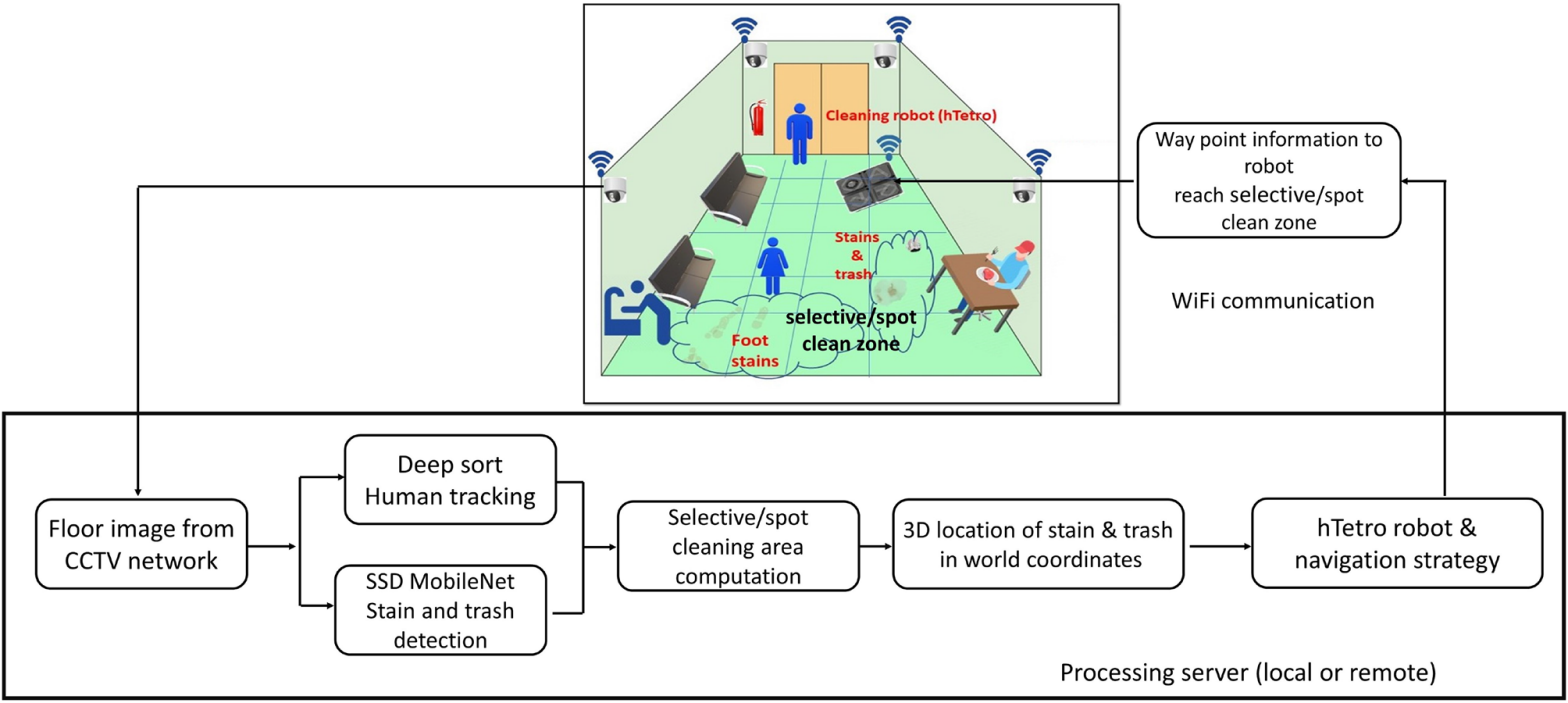
Overview of the proposed system.

Figure 1 shows the overview of our selective area cleaning/spot cleaning framework with a functional block diagram. It is developed for floor cleaning robot indoor selective area cleaning/spot cleaning task. Generally, high human traffic areas are more likely to accumulate more trash and foot stains. Hence, two strategies are combined to efficiently find the indoor environment’s selective area cleaning/spot cleaning regions. It includes tracing the human traffic patterns and detection of stains, foot stains, and trash on the floor.

The proposed system functional block diagram is composed of an image data collection module from the RGB-D vision sensor network, deep learning-based computer vision algorithms, and an optimal path planning module for the hTetro robot. WiFi-enabled surveillance camera setup (Realsense D345 fitted in roof corners) captures human movements indoors and continuously captures the floor images. The camera system is fixed in the corners of the roof, and the Field of View (FoV) is calibrated with the 3D map built by the hTetro robot base frame. The cameras are connected to an external server through a WiFi router to transfer the captured video stream’s. A deepSORT human tracking algorithm was used to trace the human traffic pattern. SSD Mobilenet object detection algorithm was trained to detect foot stains on the high human traffic region, common stains, and trash on the floor. The selective area cleaning/spot cleaning algorithm is run on an external server built with a high-performance GPU. The server collects the indoor images continuously through a WiFi router and performs the human traffic pattern detection analysis, stains, and trash detection task. According to the human traffic pattern tracing, stains, and trash detected on the floor, the framework generates the navigation strategy and send the way point coordinates to hTetro robot to perform the selective area cleaning/spot cleaning task. The robot uses waypoint information (computed through image coordinate to robot footprint coordinate) and odometry data to reach the selective area cleaning/spot cleaning location and perform the cleaning task. The details of each component and integration methods are described as follows.

### Deep SORT human tracking

The Deep SORT is an improved version of the Simple Online and Real-time Tracking (SORT) algorithm. Generally, deep sort is used for human tracking applications. In our work, Deep SORT is used to monitor the human traffic pattern tracing in indoors through RGB-D vision sensor collected image and map the high traffic area to identify the selective area cleaning/spot cleaning region. The Deep SORT tracking framework was built using the hypothesis tracking technique with the Kalman filtering algorithm and DL-based association metric approach. Further, the Hungarian algorithm is utilized to resolve the uncertainty between the estimated Kalman state and the newly received measuring value. The tracking algorithm uses the appearance data to improve the performance of sort^42^. In this work, the bounding box coordinates in image plane (X$_{min}$,Y$_{min}$ and X$_{max}$ and Y$_{max}$) of human detection are passed to Deep SORT with image frame for tracking the human movements. According to bounding box coordinates and object appearance, deep SORT assigns each human detection ID and performs the tracking. A selective area cleaning/spot cleaning algorithm used the tracked information to compute the cleaning spot.

#### Stain and trash detection framework

**Figure 2:**
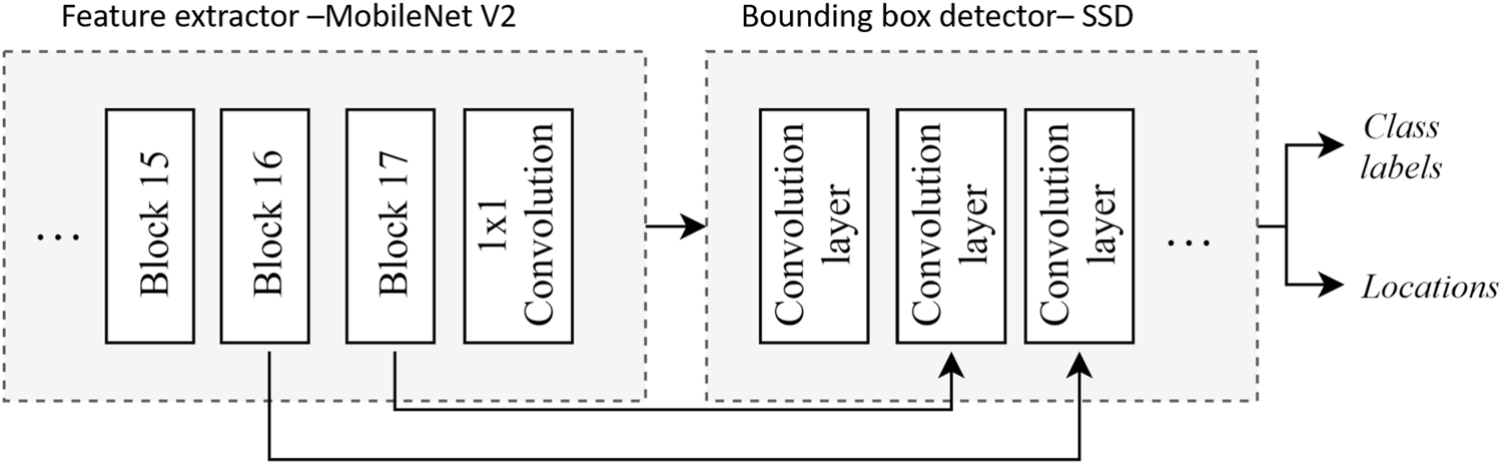
SSD MobileNet V2.

A Single Shot Detector (SSD) MobileNet V2 end-to-end object detection framework is adopted to detect the stains, foot stains, and trash on the floor. The functional block diagram of SSD MobileNet is shown in Fig. 2. Here, feature extraction is done using the MobileNet model. Bounding box prediction is made using a meta-architecture SSD. While training, RMSprop algorithm^43^ is used for loss optimization. The framework was trained using a transfer learning scheme; the last layer of the detector head is modified to detect the stains, foot stains, and trash.

#### Feature extractor

MobileNet v2 is used as the base network that performs feature extraction tasks suitable for mobile and edge devices. Figure 2 shows the MobileNet v2 architecture. It comprises multiple residual bottleneck layers. These layers use $3\times 3$ in depthwise convolution layers, which are more efficient than standard convolution layers. They also employ $1\times 1$ convolutions instead. The architecture also uses ReLU6 layers, which are ReLU layers with an upper limit of 6. The upper limit prevents the outputs from scaling to a huge value, reducing the computation cost.

#### SSD architecture

Single-Shot Detector (SSD) is an object detection algorithm. It outputs the object’s location through a bounding box in an image with the confidence of the prediction. The bounding box coordinates have been translated into real-world coordinates and fed to a trajectory planning algorithm to generate the selective area cleaning/spot cleaning path. The SSD algorithm uses the MobileNet feature map to generate the anchor boxes. The final few layers (feature map) from the feature extractor are used to predict trained objects’ locations. In the SSD architecture, the input is passed through different convolution layers of different sizes. These layers decrease in size progressively through the network. The purpose of these layers is to enable the network to detect objects of different shapes and sizes.

While training, confidence loss $L_{confidence}$ and localization loss $L_{location}$ Eq. (1) was calculated for each prediction. The prediction error of class and confidence is given by confidence loss. The localization loss is a smooth loss between the predicted bounding box value and the ground truth value. The value $\alpha$ is used to reduce the overall loss’s influence and balance confidence and location loss.

Root Mean Squared gradient descent algorithm (RMS) is used for optimizing both the losses. The weights $w_t$ are computed at any time *t* using the gradient of loss *L*, $g_t$ and gradient of the gradient $v_t$ Eqs. (2)–(4). The hyperparameter $\beta = 0.9, \eta = 0.02$ are utilized for estimating the momentum and gradient values, while a small value near to zero $\epsilon$ is used for avoiding divide-by-zero errors.

1 $$\begin{aligned} L= & {} \frac{1}{N}\left( L_{confidence} + \alpha L_{location}\right) \end{aligned}$$

2 $$\begin{aligned} v_{t}= & {} \beta v_{t-1} + (1-\beta ) g_{t}^2 \end{aligned}$$

3 $$\begin{aligned} \Delta w= & {} \frac{-\eta }{\sqrt{v_{t} + \epsilon }}\times g_{t} \end{aligned}$$

4 $$\begin{aligned} w_{t+1}= & {} w_{t} + \Delta w \end{aligned}$$

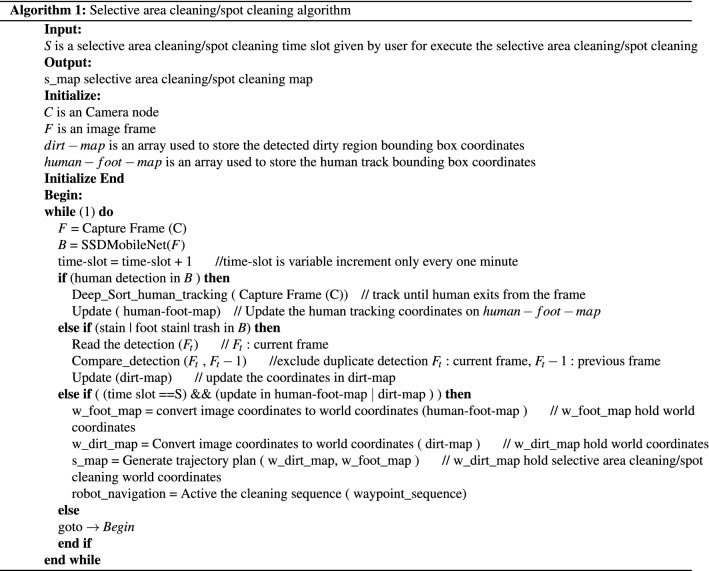


#### 3D location of stain and trash in world coordinates

After finding the 2D pixel coordinates in the RGB image frame of stain, trash, and human traffic pattern, the system will derive the world frame’s corresponding 3D position. Since, the perception system is a stereo camera with both an RGB image sensor and a depth image sensor, they are of the same resolution, and each pixel in the RGB image corresponds to the pixel in the depth image. This enables us to map the dirt object’s localized pixel coordinates to their depth value. When the depth images are subscribed, the image will be filtered via an adaptive directional filter^44^ to remove noise. After filtering the depth image, the depth value of the dirt objects, $d_o$, has been derived by Eq. (5) as follows:

5 $$\begin{aligned} d_{o}=\frac{\sum _{\Omega }d_i}{\Omega } \end{aligned}$$

where, $\Omega$ represents the sum of pixels within a $10 \times 10$ window, and $d_i$ represents the value of the depth in the pixel. The dirt objects in real-world coordinates will be estimated by converting the *X* and *Y* pixel coordinate system to *x* and *y* world coordinate system built by the Robot Lidar sensor.

RGB-D Sensor parameters are derived through camera calibration techniques to give the camera an intrinsic and extrinsic matrix^45^. Camera calibration methods give us the focal length, $f_x$, and $f_y$, of the RealSense RGB-D sensor and the optical center, $c_x$, and $c_y$, of the RealSense RGB-D sensor in its respective *x* and *y* coordinate to get the intrinsic camera matrix, *I*. The RealSense RGB-D sensor in-built functions also provide us with the translation vector, *K*, and rotation vector, *Q*. The extrinsic matrix, *S*, can be derived where $S = [Q K]$. Further, it will convert from the pixel coordinate system to the world coordinate system through Equation (6).

6 $$\begin{aligned} p = I * [Q K] * P = I * S * P. \end{aligned}$$

where *P* is the world coordinates, *p* is the pixel coordinates.

### Overview of hTetro robot and navigation strategy

**Figure 3:**
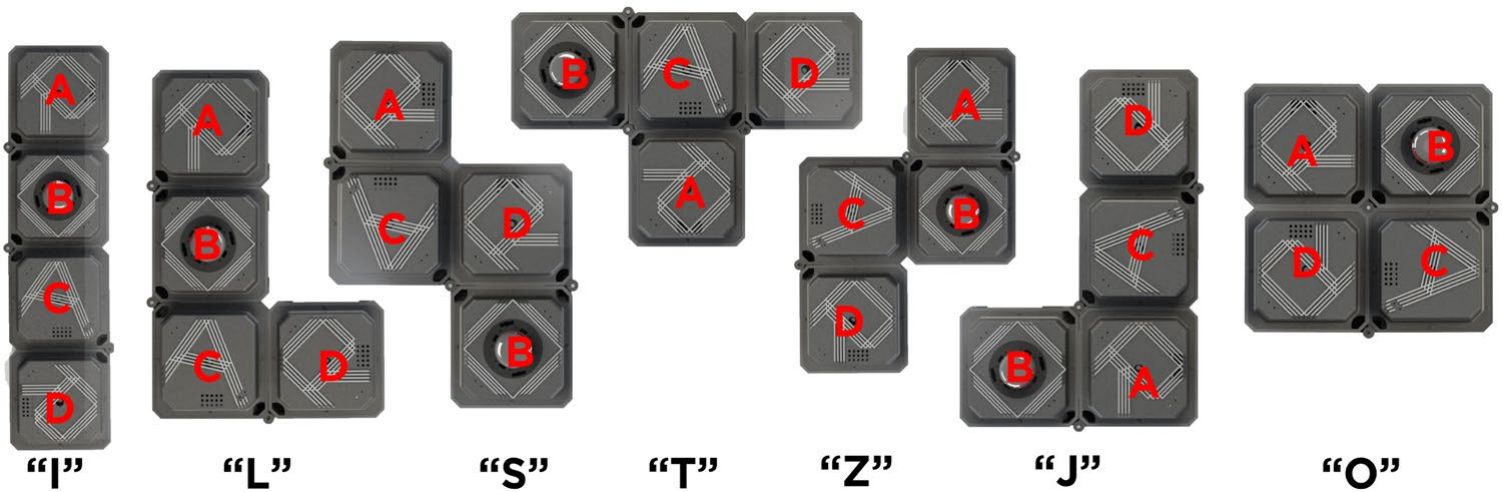
Seven configurations of hTetro.

**Figure 4:**
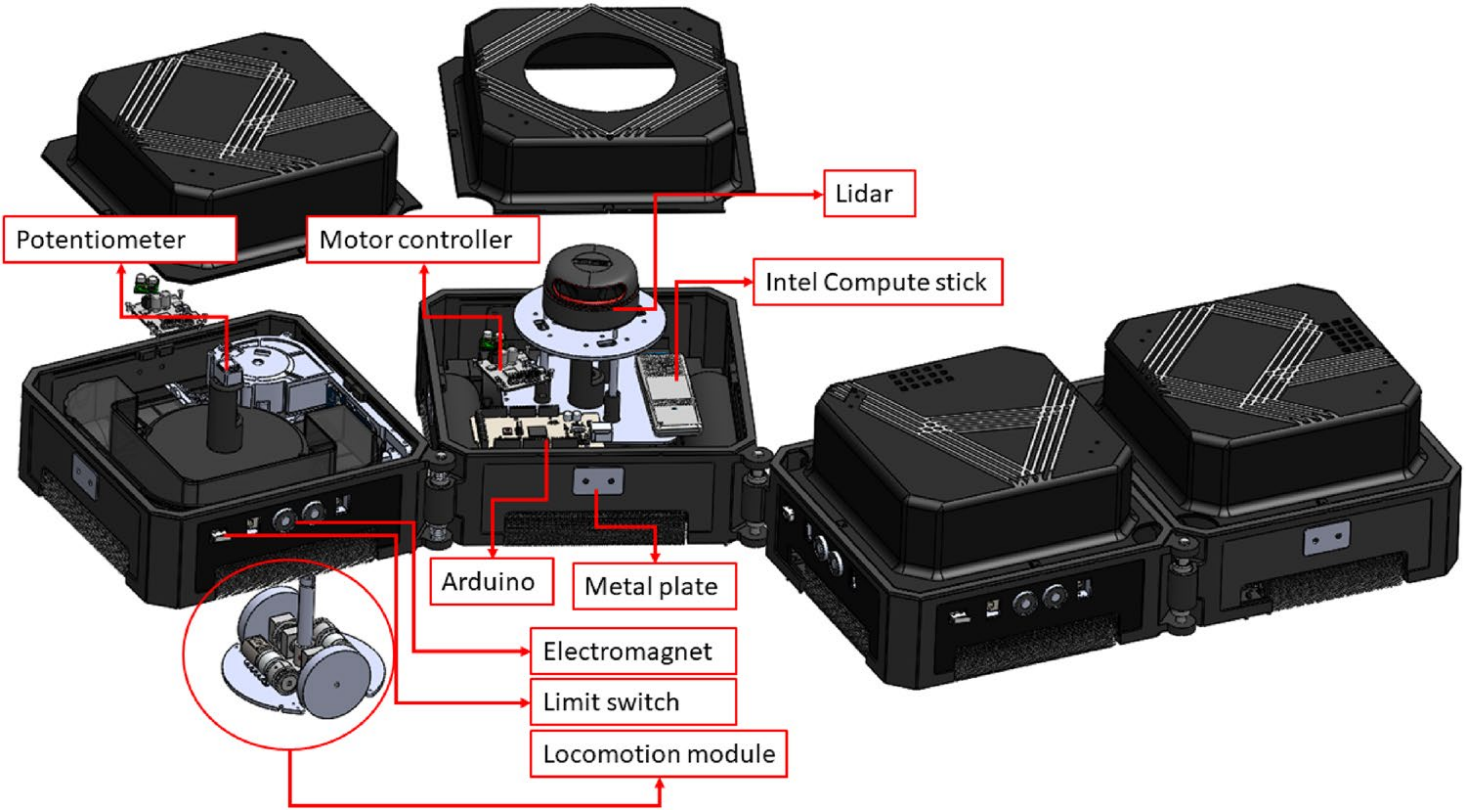
hTetro hardware components.

hTetro is an in-house developed re-configurable floor cleaning robot (Fig. 4). This robot consists of a chain-type with four cube shape modules connected by three hinges at the corner of blocks, as shown in Fig. 3. In hTetro, blocks 1, 3, and 4 each hold a cleaning module, while block 2 contains the primary electronic circuits. The robot blocks could be easy to remove and add a degree of freedom. The hinges permit the hTetro robot in shape-transferring and a couple of configurations. The shape-shifting allows the robot to cover the constrained space more efficiently than fixed-form robots. For instance, the robot can cover the bigger space with the default O shape and change shapes flexibly from the O to I shape to cover the narrow spaces. The three hinges connected between robot blocks are used for shape-shifting during the reconfiguration.

The locomotion modules at each block are programmed to operate synchronously with others according to the robot’s transformation, and each of the modules is amenable for steering and moving the hTetro. Each block contains two geared DC motors with encoders for balance locomotion. The operation voltage rating of the DC motors is 7.4V. In hTetro, a potentiometer type absolute encoder was used in each block to sense the shape change action and block’s heading angle. When the hTetro is involved in the transformation actions, the servos located at the hinges drive the modules by rotation clockwise and anticlockwise to do the reconfiguration into seven distinct shapes -**‘I, L, S, T, Z, J, O’**, as shown in Fig. 3. The low-level controller (Arduino mega) was used to measure the output current from the absolute encoder through the A/D Input pin and fine-tunes the shape change action. This sensing data helps ensure that the units are aligned and moving in the same direction. The servo motors run at 14.8V, and the high torque is 77 kg. cm. The servo has enough torque to hold the hTetro block masses in morphologically changing and fastening the blocks in locomotion.

#### Hardware and software components

hTetro hardware architecture was developed with a single-board computer Intel compute stick and low-level micro-controller Arduino mega 2560 (Fig. 4). The Intel compute stick is powered with an Intel Core m3 processor, 4GB of memory, and 64GB of eMMC flash storage. It was used as an hTetro central processing unit, runs with an operating system of Ubuntu 20.04, and uses ROS Melodic^46^ as middleware. The hTetro navigation algorithms run alongside the ROS middleware in the compute stick and control all the autonomy-related operations of the hTetro. The navigation algorithm receives the waypoint information from the remote/local server through on board WiFi module and generates the control command to the low-level control unit to execute the selective area cleaning/spot cleaning task.

Arduino-Mega was used as a low-level controller. It works at 5 volt supply voltage and operates at 16 Mhz clock frequency. The controller receives the commands from the central processing unit through ROS serial bridge function and generates the appropriate actions for the motor drives and other local electronic components. The rosserial ROS package uses Arduino’s universal asynchronous receiver/transmitter (UART) communication and converts the board to a ROS node that can output ROS messages and subscribe to messages as well.

#### Optimization of trajectory

The 3D points at the centers of detected objects are defined as waypoints the robot needs to visit to do the appropriate selective area cleaning/spot cleaning region. The robot will derive the optimal navigation trajectories defined as Complete Waypoint Coverage Planning (CWCP) to visit all the waypoints. With *N* unsequenced waypoints detected from the perception unit, the navigation trajectory $\eta$ is defined as follows: $\eta ~=~\{(W_1^s, W_1^g), (W_2^s, W_2^g) ..., (W_N^s, W_N^g)\}$ The trajectory $\eta$ consists of pairs of waypoints with the directions which indicate the order of navigation. The initial waypoint is labeled $W_1^s$, and the final waypoint is labeled $W_N^g$. This trajectory representation visits each waypoint among *N* waypoints once. The cost to navigate from source waypoint to destination waypoint equals to summation of mass of each robot $m_i$ block multiplied with 3D displacement to move blocks from source $(x^s_i,y^s_i,z^s_i)$ to goal $(x^g_i,y^g_i,z^g_i)$ as shown in Eq. (7). The total cost of the trajectory can be summarized by all the waypoint pair components as shown in Eq. (8). The Pareto trajectory is defined as $\widehat{\eta }$, and the objective of the proposed CWPP problem is derived by Eq. (9).

7 $$\begin{aligned} C \left( W_n^s, W_n^g \right)= & {} \sum _{i=1}^{4}m_i\sqrt{\left( x^g_i-x^s_i \right) ^2+\left( y^g_i-y^s_i \right) ^2 +\left( z^g_i-z^s_i \right) ^2} \end{aligned}$$

8 $$\begin{aligned} C_{total}= & {} \sum _{\left( W^s_n,W^g_n \right) \in \eta }^{}C \left( W_n^s, W_n^g \right) \end{aligned}$$

9 $$\begin{aligned} \hat{\eta }= & {} \underset{\eta }{argmin} \, C_{total}= \underset{\eta }{argmin}\sum _{\left( W^s_n,W^g_n \right) \in \eta }^{}C \left( W_n^s, W_n^g \right) \end{aligned}$$

There are many possibilities to form the trajectories and find the optimal route to minimize the fitness function; the classic Travelling Salesman Problem (TSP) model can be considered an NP-hard problem. Similarly, brute force search can be used for iterating every possible path with factorial time complexity O(*n*!), But performance gradually decreases with the large input data size. Dynamic programming through Held-Karp algorithm^47^ reduces the complexity of TSP to O($n^22^n$) time. However, the method is still inappropriate for the presented scenario where multiple detected waypoints are in the working workspace. Evolutionary approach Ant Colony Optimization (ACO)^48,49^ is adopted to find the optimal trajectory connecting the waypoints, $\hat{\eta }$ in Eq. (9) within a reasonable time. Optimization based on probabilistic techniques are the main idea of ACO. By tuning the ant colony decisions at the waypoint and the constant updates on the pheromones left along the temporary trajectory, the ACO algorithm has proven to be a feasible method to derive the Pareto-optimal solution of the TSP while eliminating the calculation burden.

## Experimental results

This section presents the experimental procedure and outcome of the selective area cleaning/spot cleaning framework tested on the hTetro robot. The algorithm has two pre-processing steps: training the trash and stain detection framework using a custom dataset and mapping the indoor environment for hTetro autonomous navigation.

The dataset was prepared in-house with different image resolutions. The dataset has four classes (Stain, Foot stain, Trash and Human); each class has 1200 images. Most of the images are created in-house with various floor backgrounds, and few images are collected online through bing image search. Here, in-house collected images are captured in an overhead perspective with a Realsense RGBD camera with a different angle and different illumination conditions. Then, the captured images are labeled for training the detection framework (SSD MobileNet). Further, to reduce the CNN learning rate and avoid over-fitting. The data augmentation (rotation, scaling, and flipping) is applied in collected images.

The standard method of the K-fold (here K = 10) cross-validation process is used to evaluate the detection model. In K-fold cross-validation, the labeled images are split into ten sets, and nine sets were used to train the detection network, and one set is utilized for evaluation. These steps run for ten rounds to ensure all the data were used for both training and testing to eliminate the biasing conditions. This process is done to remove any bias due to the particular training and testing data set to split. The experimental results are obtained from the detection model with reasonable accuracy. The deep learning models were developed in Tensor-flow 1.9 Ubuntu 18.04 version and trained using the following hardware configuration Intel core i7-8700k, 64 GB RAM, and Nvidia GeForce GTX 1080 Ti Graphics Cards. Standard statistical measures such as accuracy (10), precision (11), recall (12) and $F_{measure}$ (13) were used to evaluate the proposed algorithm through confusion matrix elements.

10 $$\begin{aligned} Accuracy (A)= & {} \frac{tp + tn}{tp + fp + tn + fn} \end{aligned}$$

11 $$\begin{aligned} Precision (P)= & {} \frac{tp}{tp + fp} \end{aligned}$$

12 $$\begin{aligned} Recall (R)= & {} \frac{tp}{tp + fn} \end{aligned}$$

13 $$\begin{aligned} F_{measure} (F_{1})= & {} \frac{2 \times precision \times recall}{precision + recall} \end{aligned}$$

Here, *tp*, *fp*, *tn*, *fn* represents the true positives, false positives, true negatives, and false negatives, respectively, as per the standard confusion matrix.

### Validation of selective area cleaning/spot cleaning

**Figure 5:**
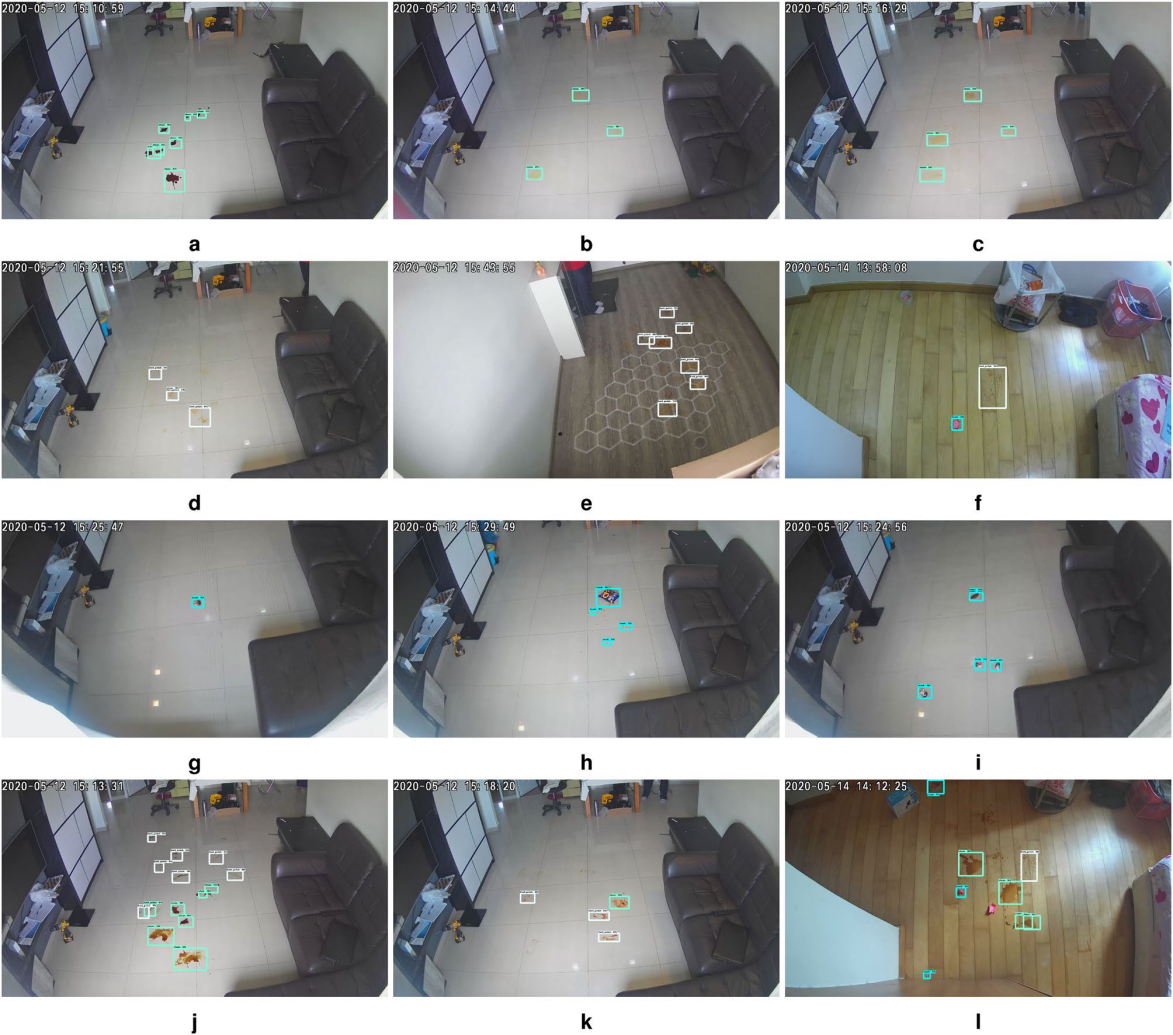
Real-time detection of stain, foot stain, trash.

**Figure 6:**
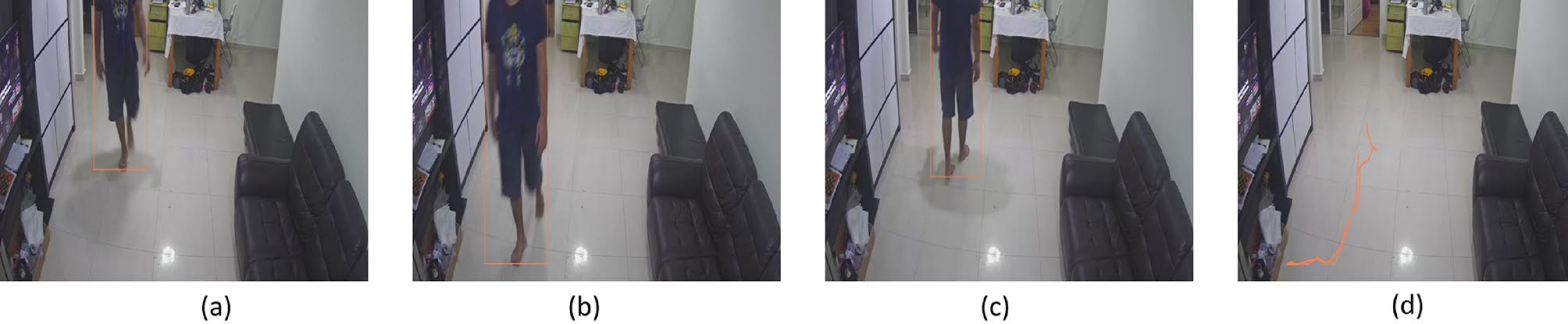
Human movement tracking, (**a**–**c**) human movements and (**d**) tracking result.

The selective area cleaning/spot cleaning framework was validated in three different indoor environments with the sizes of $10 \times 10, 8 \times 8, 15 \times 8$ feet. The overhead cameras were fixed corner of the roof in the testbed for tracing the human movements and capturing the floor images. The captured floor images are transferred to the external server, running a selective area cleaning/spot cleaning framework. The experiment was carried out with different lighting conditions, and results are shown in Figs. 5 and 6. In this trial, the overhead camera run at 30fps, and the image resolution was $640\times 480$ pixels. The floor image was captured at 7 feet height from the ground, and the camera head is inclined at an angle of 45 degrees downwards.

The stain is detected by a light turquoise color bound box, foot stains were marked as white rectangle box and trash marked by the sky blue bounding box. Figures 5 and  6 shows the human traffic pattern detection results. Here, the human movements were tracked by bounding boxes, and the traffic pattern is indicated in the orange line. The experiment results indicate that the selective/ spot cleaning framework detects the human, stain, and trash with a higher confidence level of $92\%$, $90\%$, 95%, and $98\%$, respectively. Further, the model detection accuracy was estimated by statistical measures, which are given in Table 2.

**Table 2:** Statistical measure results.

Class	P	R	F1	A
Stain	93.07	92.0	91.15	93.44
Foot stain	92.11	90.22	90.04	91.69
Trash	94.55	93.89	93.32	93.0
Human	97.02	96.3	96.03	97.0

The statistical measurement result shows that the framework has identified the stain with an average of 93.44%, foot stains with 91.69%, trash with 93.0 %, and human with 97.0%, respectively. In this experiment, we observe that stain and foot stain detection confidence level and statistical measure values are relatively low compared to other classes due to less visibility of objects and complex texture shape. Further, the computation cost of the proposed system was estimated via inference time; in this test, the framework took 0.05 seconds to process the $640 \times 480$ images. The statistical study ensures that the proposed system was stable for different lighting conditions and also work in realtime.

### Comparison with other object detection frameworks

The efficiency of the SSD mobilenet stain trash detection was evaluated with other benchmark object detection algorithms include Faster RCNN ResNet^50^, Faster RCNN Inception^51^, and Yolo v2 frameworks. The same dataset was used for estimating the detection and classification accuracy of three networks for a period at the same time. Statistical measure parameter was used to compare the performance model. Table 3 shows statistical measure results of each framework. In addition to that, timing analysis was performed to calculate the detection time taken by each network. NVIDIA Quadro P4000 graphic card was used for testing the proposed algorithm, as shown in Table 4.

**Table 3:** Comparison with other object detection frameworks.

Class	Faster RCNN ResNet				Faster RCNN Inception				Yolo V2			
	P	R	$F_{1}$	A	P	R	$F_{1}$	A	P	R	$F_{1}$	A
Stain	95.96	94.34	94.11	95.04	94.41	94.38	94.11	94.0	89.24	88.11	87.66	89.15
Foot stain	94.45	94.13	94.01	94.24	93.50	92.07	92.06	92.00	85.00	84.33	84.09	84.50
Trash	96.63	96.29	96.17	96.44	96.20	95.91	95.43	96.03	89.04	89.25	89.06	90.00
Human	97.35	96.92	96.74	97.00	96.87	96.43	96.14	96.54	93.01	91.25	91.03	92.00

**Table 4:** Execution time comparison.

Model	Inference time for 60 images (s)
Faster RCNN ResNet	122
Faster RCNN Inception	110
Yolo V2	48
SSD MobileNet	54

Best detection accuracy was obtained using Faster RCNN ResNet and Faster RCNN inception compared to other models. Yolo v2 inference time is comparatively low but showed poor detection accuracy. SSD-MobileNet has obtained a better trade-off between detection accuracy and inference time. Hence, SSD- MobileNet shows optimum results when used in realtime applications for object detection. Optimized SSD MobileNet is highly suitable for low-power resource-constrained embedded systems, making it suitable for realtime applications.

### Complete Waypoints Coverage Planning (CWCP) evaluation

We validated the efficiency and performance of the selective area cleaning/spot cleaning method in terms of navigation efficiency by deploying the cleaning robot system to clean optimally the detected tracked human traces in the selected indoor testbed sites. Specifically, depending on the human interaction activity in the environment, a location map is constructed on the grid cells by the proposed framework. The location map distribution result is used as input for a hTetro reconfigurable cleaning robot to decide navigation strategies optimally.

**Figure 7:**
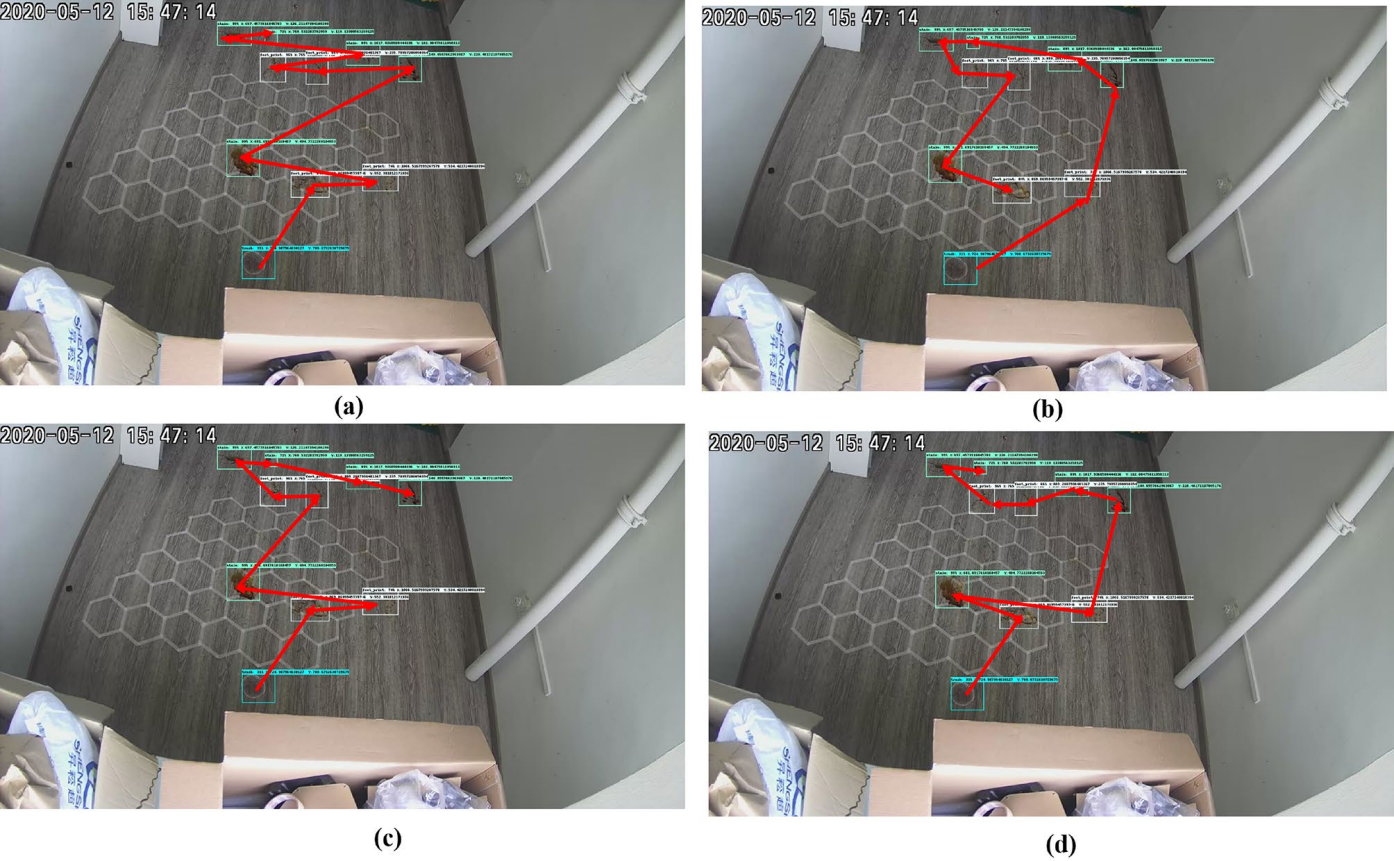
The trajectories of the tested method for workspace-1. (**a**) zigzag; (**b**) spiral; (**c**) greedy search; (**d**) ACO.

**Figure 8:**
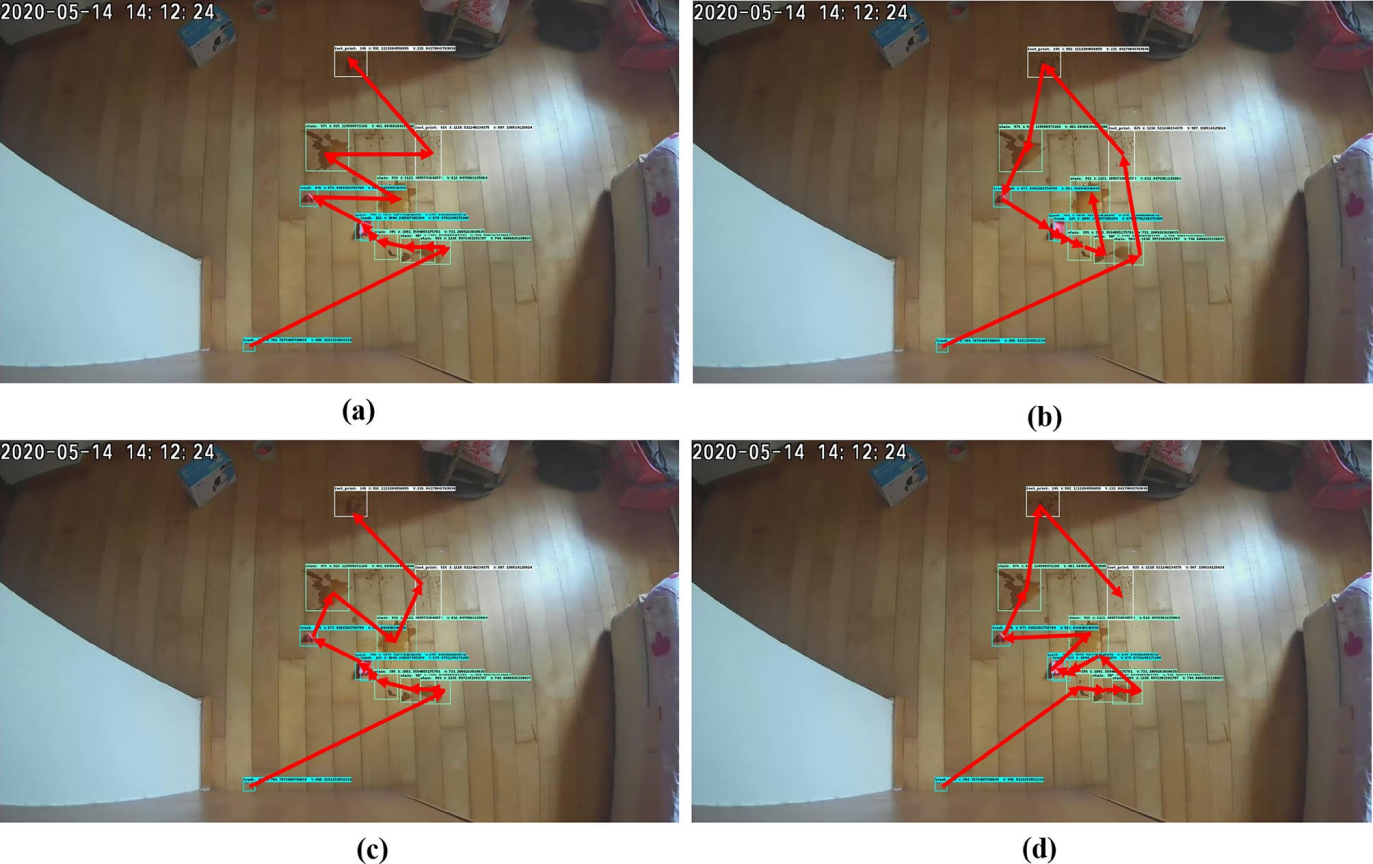
The trajectories of the tested method for workspace-2. (**a**) zigzag; (**b**) spiral; (**c**) greedy search; (**d**) ACO.

The Complete Waypoints Coverage Planning (CWCP) framework derives the smallest costweight and energy consumption for real environments testbed. The trajectories denoted as red linking arrows generated by all teed methods including zigzag, spiral, greedy search with 100 iterations, and ACO algorithms for all testbed workspace-1 with 9 detected waypoints and workspace-2 with 11 detected waypoints are shown in Figs. 7 and 8, respectively. In the experiment, the best parameter values are found by the experimental approach by 200 trials. after tunning, the ACO parameters set is evaporation probability = 0.9, and ant agents = 220. The terminal’s criteria are triggered when the costweight during execution is not improved for ten iterations or the execution loop runs over the 500 iterations.

The trajectories with found costweight and the time to generate as well as the running time to complete these found trajectories of the tested method for the two testbed workspaces are represented in Table  5, respectively. Because of the straightforward approach in generating the paths, the path generation time of the zigzag and the spiral are smaller than Greedy Search and ACO, but the zigzag and spiral methods link the defined locations by position-wise orders; hence the costweight is greater than greedy search methods and significantly greater than ACO based Complete Waypoints Path Planning (CWPP). Noted that a greedy algorithm is an algorithm that follows the problem-solving heuristic of making the locally optimal choice at each stage. Since it may be stuck at local minimal so we run the greedy algorithm for 100 iteration with random beginning waypoint and select the best solution. That makes that greed search requires more path generation time.

In terms of running time when deploying the found trajectory to the real robot, the Zigzag and Spiral need more time to cover the defined waypoint than the greedy search. Together with the least costweight, the running time of ACO-based CWPP is the shortest one.

**Table 5:** The numerical comparisons for tested CWPP methods.

Approach	Cost weight (Kgcm)	Consumed energy (Wh)	Path generation time (s)	Running time (s)
Zigzag	1951.69	0.014344444	0.01	182.25
Spiral	1962.28	0.016172222	0.05	194.16
Greedy search	1829.63	0.010866667	5.25	135.95
ACO	1725.91	0.0098916667	0.92	125.15

After the perception system has defined the waypoint locations in real testbed sites, the energy efficiency during the selective area cleaning/spot cleaning process is validated by recording in realtime the energy consumption. The current sensors connected to the main lithium-ion battery with the specs of 14.4 V, 3000 mAh are logged during robot operation. These current values are integrated with the running time to find power consumption. In the selective area cleaning/spot cleaning mode, the hTetro is set at O shape and to clear these waypoints in order. The robot can change to I shape to access the narrow space area by the environment evaluation from the Lidar sensor. The action of shape-changing is adapted to a dynamic environment. The robot modules such as perception, locomotion, and shape-shifting communicate under the controls by the ROS system^52^. To archive the smooth locomotion with the different kinematic design of hTetro, the moving command generated by the PID controller is sent to motor drivers to issue appropriate velocity for the DC motors. Specifically, the robot should keep the planned trajectories and overcome the disturbances while colliding with static and dynamic obstacles. These tasks are significant challenges since the synchronization between hTetro blocks locomotion must be accomplished. To fulfill the kinematic constraints, the control method is based on the Instantaneous Center of Rotation (ICR). The placement of ICR makes use of the desired positions and velocities information provided by the central computing unit based stains pattern. After that, the corresponding steering angle and required individual velocity are calculated based on the ICR location. A velocity regulator is then used to limit the velocity of each motor. Due to the placement of LIDAR on the second module’s chassis, the orientation of the whole robot is defined according to it. The instantaneous center of rotation’s desired location is defined based on the desired position, current position, and the desired velocity. It is represented in the polar coordinate using to avoid numerical singularity. The hTetro locates itself within the pre-built map using enhanced extended Kalman filter(EKF)^53^ for multi-sensor fusion makes sure hTetro can navigate from source and destination goad correctly even if sensors noise is received, or any perception sensors struggle the malfunction issues.

From Table  5, if the robot clears the found path of the method with a smaller costweight value, the less energy spent is observed. Specifically, since producing the longest costweight, the zigzag draws the highest energy, then the spiral follows the following position with a slight amount lower. The best CWCP method in both energy and time saving is ACO. It yields about 10% and 15% lower than the greedy search in energy and time spent, respectively. The results ensure that the proposed CWCP with ACO is possible for selective area cleaning/spot cleaning by hTetro.

## Conclusions

This work proposed a selective area cleaning/spot cleaning technique for reconfigurable floor cleaning robot hTetro using deep learning-based computer vision algorithms and an optimal complete waypoints path planning method. SSD MobileNet object detection algorithm and Deep SORT real-time human tracking algorithm were used to find the human traffic patterns and detect the stains, foot stains, and trash on the floor. The proposed scheme was tested with two different testbeds with hTetro reconfigurable floor cleaning robot and evolutionary optimization based optimal trajectory generation algorithm. The experimental results showed that the trained SSD model detects the stains, foot stains, and trash on the floor with a high confidence score, and its result is used to find the selective area cleaning/spot cleaning region. Further, the SSD MobileNet framework’s stain and trash detection efficiency was evaluated with other CNN-based object detection algorithms and observed that SSD MobileNet obtained a better trade-off between detection accuracy and low inference time for detection of stains and trash. The overall experimental results show that the proposed framework is more suitable for enabling the autonomous selective area cleaning/spot cleaning function for the reconfigurable floor cleaning robot hTetro.

## Data Availability

‘The datasets used and/or analysed during the current study available from the corresponding author on reasonable request.’ Correspondence and requests for materials should be addressed to B.R.
